# Cognitive function enhancement in Alzheimer’s disease through traditional Chinese medicine rehabilitation nursing: meta-analysis

**DOI:** 10.3389/fpsyt.2025.1631589

**Published:** 2025-08-13

**Authors:** Jinping Zhao, Xiaohong Dong, Bin Liu, Yubo Peng, Zhuang Yao

**Affiliations:** ^1^ Liaoyuan Vocational and Technical College, Liaoyuan, Jilin, China; ^2^ Nursing Department of Jiamusi College, Heilongjiang University of Traditional Chinese Medicine, Jia Musi, Heilongjiang, China; ^3^ Basic medical school of Heilongjiang University of Traditional Chinese Medicine, Harbin, Heilongjiang, China

**Keywords:** traditional Chinese medicine, cognitive rehabilitation, Alzheimer’s disease, dementia, meta-analysis, nursing intervention

## Abstract

Alzheimer’s disease (AD) manifests as progressive cognitive deterioration with significant impact on patient independence and quality of life. While conventional treatments offer limited efficacy, Traditional Chinese Medicine (TCM) rehabilitation nursing presents a complementary approach deserving systematic evaluation. To synthesize existing evidence on the efficacy of TCM rehabilitation nursing for cognitive enhancement in AD through comprehensive meta-analysis. We conducted systematic searches across multiple electronic databases (PubMed, Embase, CNKI, Wanfang, and VIP) for controlled studies published from 2010 to present examining TCM rehabilitation nursing interventions for AD patients. Methodological quality was assessed using the Cochrane Risk of Bias 2.0 tool. Primary outcomes included Mini-Mental State Examination (MMSE), Activities of Daily Living (ADL), and treatment efficacy rates. Statistical synthesis employed RevMan 5.3 with random or fixed effects models based on heterogeneity assessment. Nine eligible studies encompassing 864 participants (432 intervention, 432 control) met inclusion criteria. Meta-analysis revealed significantly improved cognitive function in the TCM rehabilitation nursing group compared to conventional care, with MMSE scores showing substantial enhancement (mean difference = 4.63, 95% confidence interval: 3.74-5.53, P<0.00001). Treatment response analysis demonstrated higher rates of marked clinical improvement (risk ratio = 2.78, 95% CI: 1.65-4.70, P=0.0001) and substantially reduced treatment failure rates (85% reduction, P<0.00001). Though ADL scores showed positive trends, these did not reach statistical significance (P=0.07). TCM rehabilitation nursing demonstrates significant efficacy in enhancing cognitive function and treatment outcomes in AD patients. These findings support its integration into comprehensive care strategies, though additional research with standardized protocols is warranted for optimal implementation.

## 1 Introduction

Alzheimer’s disease (AD) represents a progressive neurodegenerative disorder characterized by gradual cognitive deterioration, functional decline, and behavioral changes. The clinical manifestations typically include memory impairment, language difficulties, executive dysfunction, visuospatial deficits, and alterations in personality (1). Within the spectrum of neurodegenerative conditions affecting older adults, AD stands as particularly prevalent, emerging as a significant global health challenge that demands increased scientific and clinical attention (1).

Epidemiological investigations reveal concerning trends in AD prevalence and projection. Current global estimates indicate over 47 million individuals affected by this condition. The demographic shift toward an aging population suggests this number may approach 131 million by mid-century (2). This phenomenon presents particular challenges for countries experiencing rapid population aging, such as China, where AD prevalence has surpassed 5% among elderly citizens (3).

In China, the elderly population (aged 65 and above) reached 190.6 million in 2020, accounting for 13.5% of the total population, and is projected to grow to 394 million by 2050, representing 30.2% (4). From 1980 to 2020, the percentage of adults aged 60 years and older increased from 6.9% to 18.7%, reaching 264 million people, driven by urbanization and migration (5). The number of people living with dementia in China is expected to triple from 16.3 million in 2020 to 49 million by 2050 (4).

Despite decades of intensive research efforts, the precise pathophysiological mechanisms underlying AD remain incompletely understood, complicating treatment development. These gaps in mechanistic understanding have hindered the development of disease-modifying therapeutics, leaving clinicians to rely heavily on symptomatic management and supportive care approaches. This therapeutic limitation underscores the importance of exploring complementary strategies that may offer cognitive benefits even without addressing the underlying pathology. The mortality impact of AD cannot be overstated, as it currently ranks fourth among leading causes of death globally, following cardiovascular disease and cerebrovascular events (6). This mortality burden, combined with the prolonged disease course and progressive functional decline, creates substantial challenges for healthcare systems and caregivers alike. The extended trajectory of cognitive deterioration particularly necessitates innovative approaches to maintain functional capacity and quality of life throughout the disease continuum.

Clinical management strategies for AD patients frequently incorporate specialized nursing interventions designed to modify disease progression and optimize functional capacity (7). These interventions typically focus on cognitive stimulation, environmental modification, and caregiver education. However, conventional approaches often demonstrate limited efficacy in maintaining cognitive function over extended periods, prompting investigation into complementary and alternative methodologies that might offer additional therapeutic benefits.

Traditional Chinese Medicine (TCM) rehabilitation nursing represents one such complementary approach, frequently employed as an extended therapeutic strategy for AD management (8, 9).TCM rehabilitation nursing is a holistic, multidimensional approach grounded in TCM principles, integrating psychological and physical modalities such as acupuncture, acupressure, massage, Qi Gong (meditation-based treatment), herbal supplementation, dietary guidance, and rehabilitation exercises to restore balance (yin-yang harmony) and energy flow (qi), rather than isolated symptom management. Its potential efficacy in AD stems from addressing interconnected domains (cognitive, emotional, physical), potentially enhancing cerebral circulation, reducing harmful metabolites, and improving quality of life through non-pharmacological means, as supported by applications in related conditions like stroke (8–11).

Implementation of these specialized nursing protocols potentially enhances therapeutic outcomes while simultaneously improving quality of life metrics and long-term prognosis for affected individuals (9). Chinese patients often prefer TCM for dementia due to its long history in improving memory and cognitive function, multi-target approach (addressing multiple pathways unlike single-target Western drugs), fewer side effects, and cultural integration as a primary or adjuvant therapy. Clinical trials in China have shown TCM improving cognitive outcomes, making it a valued option for preventing and treating AD (12).

Evidence supporting TCM rehabilitation nursing efficacy extends beyond AD applications. For instance, Xie and colleagues demonstrated that TCM-based rehabilitation nursing combined with scalp acupuncture significantly improved emotional status and quality of life parameters among stroke survivors (10). Similarly, Dai et al. reported substantial improvements in symptomatology, quality of life metrics, and physiological indicators when traditional Chinese rehabilitation nursing techniques were applied to chronic heart failure patients (11). These findings across diverse medical conditions suggest potential transferability of TCM rehabilitation principles to cognitive disorders, though the specific mechanisms and magnitude of effect may differ substantially based on underlying pathophysiology and symptom presentation. The holistic nature of TCM approaches may address multiple aspects of AD simultaneously, potentially offering advantages over more narrowly focused conventional interventions. Despite these promising applications in various medical conditions, systematic evaluation of TCM rehabilitation nursing specifically for cognitive enhancement in AD populations remains notably underdeveloped. While individual studies have reported positive outcomes, the absence of comprehensive synthesis limits clinical implementation and policy development. Furthermore, significant methodological variations across studies complicate interpretation of efficacy and implementation protocols, highlighting the need for rigorous meta-analytical approaches to consolidate existing evidence. The present investigation undertakes a systematic search and meta-analytical examination of literature documenting cognitive function modification through TCM rehabilitation nursing interventions for AD patients. Through rigorous analysis of relevant research, this study aims to quantify the cognitive impact of TCM rehabilitation nursing approaches, ultimately contributing to the development of evidence-based rehabilitation protocols for this vulnerable population.

## 2 Data and methods

### 2.1 Study design and search strategy

This meta-analysis followed the Preferred Reporting Items for Systematic Reviews and Meta-Analyses (PRISMA) guidelines. We conducted comprehensive searches across multiple electronic databases including PubMed, Embase, China National Knowledge Infrastructure (CNKI, https://www.cnki.net/), Wanfang Database and VIP database for relevant publications from 2010 to present. Search terminology encompassed various combinations of: (“Alzheimer’s disease” OR “AD” OR “dementia”) AND (“Traditional Chinese Medicine” OR “TCM”) AND (“rehabilitation nursing” OR “nursing intervention”). Controlled descriptors included MeSH terms such as “Alzheimer Disease,” “Medicine, Chinese Traditional,” “Rehabilitation Nursing.” For international database searches, we employed terms including AD and Individual recognized training. Database exploration was performed independently by two researchers between January 2023 and March 2023. Additionally, reference lists from identified publications were manually examined to capture any overlooked relevant studies. Full search strategies for each database are provided in **Supplementary File**.

### 2.2 Selection criteria

For study inclusion, we established the following requirements:

1. Research design: exclusively randomized controlled trials.
2. Population characteristics: subjects with confirmed AD diagnosis per International Classification standards, without comorbid conditions.
3. Intervention comparison: experimental groups receiving TCM rehabilitation nursing (integrated modalities like acupuncture, massage, dietary guidance, and emotional regulation) versus control groups under standard nursing care (conventional practices focused on symptomatic management, safety, and support, including thorough history/physical exams, neurological/psychiatric assessments, mood/behavior evaluation, nutrition/dressing ability checks, assigning consistent staff, avoiding room changes, and pain/discomfort assessment).
4. Assessment parameters: measures reflecting cognitive status through self-reporting or objective evaluation.
5. Reportable outcomes: comprehensive data on MMSE scores (13, 14), ADL measures (14), treatment efficacy rates (significant, effective, ineffective), with complete quantifiable datasets.

Studies were excluded based on these parameters:

1. Methodologies lacking proper randomization protocols.
2. Investigations with insufficient or fragmentary data reporting.
3. Duplicate or redundant publications.
4. Research examining AD with concurrent pathologies (stroke, cardiovascular conditions, etc.).
5. Non-empirical works including reviews, conference abstracts, or inaccessible full-text documents.
6. Investigations lacking sufficient statistical parameters for effect size calculations.

### 2.3 Data extraction and quality evaluation

Independent examination of eligible studies was performed by two investigators utilizing Revman5.3 templates for systematic data extraction. Cross-verification of extracted information occurred between researchers, with discrepancies resolved through consultation with a third investigator.

The extraction process captured: (1) Publication identifiers including authorship, publication timeframe, and participant numbers; (2) Subject demographics, allocation details, and intervention specifications; (3) Primary and secondary outcome measurements. We documented extensive details regarding TCM rehabilitation nursing protocols, specifically noting technique variations (acupuncture approaches, massage methodologies, emotional regulation strategies, nutritional guidance), treatment scheduling and duration parameters, and practitioner qualifications when such information was available. Comparable documentation was maintained for control interventions to establish clear comparative frameworks. For studies reporting participant attrition, we noted whether intention-to-treat (ITT) analytical approaches were employed. When data clarification was necessary, corresponding authors were contacted for additional information. Quality assessment employed the (15)Cochrane Risk of Bias tool version 2.0 (RoB 2.0) (16). Two independent reviewers assessed domains (randomization process, deviations from intended interventions, missing outcome data, measurement of outcomes, selection of reported results) using RoB 2.0 signaling questions and algorithm, with overall bias judged as low, some concerns, or high. Disagreements were resolved by a third reviewer.

### 2.4 Outcome measures

Our primary assessment parameter focused on cognitive performance as evaluated through the Mini-Mental State Examination (MMSE), a validated cognitive screening instrument that evaluates multiple domains including orientation, attentional capacity, memory function, linguistic abilities, and visual-spatial processing, scored on a 30-point scale with higher values indicating superior cognitive capability.

Secondary assessment parameters included:

1. Functional independence measured through Activities of Daily Living (ADL) scoring systems.
2. Therapeutic response categorized hierarchically as:

- Significant efficiency (substantial improvement in symptomatology and cognitive parameters).
- Effective efficiency (moderate symptomatic and cognitive enhancement).
- Inefficiency (minimal or absent clinical improvement).

These classification frameworks derived from standardized evaluation criteria reported within the original investigations and represent graduated levels of clinical response to therapeutic intervention.

### 2.5 Statistical analysis

Statistical processing employed Revman 5.3 software from the Cochrane Collaboration platform. Heterogeneity assessment incorporated manual evaluation techniques and χ^2^ testing. When minimal heterogeneity was detected (P≥0.1, I^2^ ≤50%), fixed-effect modeling was implemented; conversely, substantial heterogeneity (P<0.1, I^2^>50%) necessitated random effects modeling approaches. For continuous variables, weighted mean difference (WMD) calculations generated standardized mean differences with corresponding 95% confidence intervals (CI). Dichotomous outcomes (significant efficiency, effective efficiency, and inefficiency) were analyzed using risk ratio (RR) calculations with 95% CI parameters. To assess result stability, sensitivity analysis involved sequential study exclusion with recalculation of pooled effect measurements. Exploratory subgroup analyses were conducted for heterogeneity exploration using standardized mean differences (Hedges’ g) where data allowed. Publication bias evaluation incorporated funnel plot visualization with statistical significance established at P<0.05. Quantitative publication bias assessment employed Egger’s test methodology when sufficient studies were available for meaningful analysis.

## 3 Results

### 3.1 Study selection and characteristics

**Figure 1:**
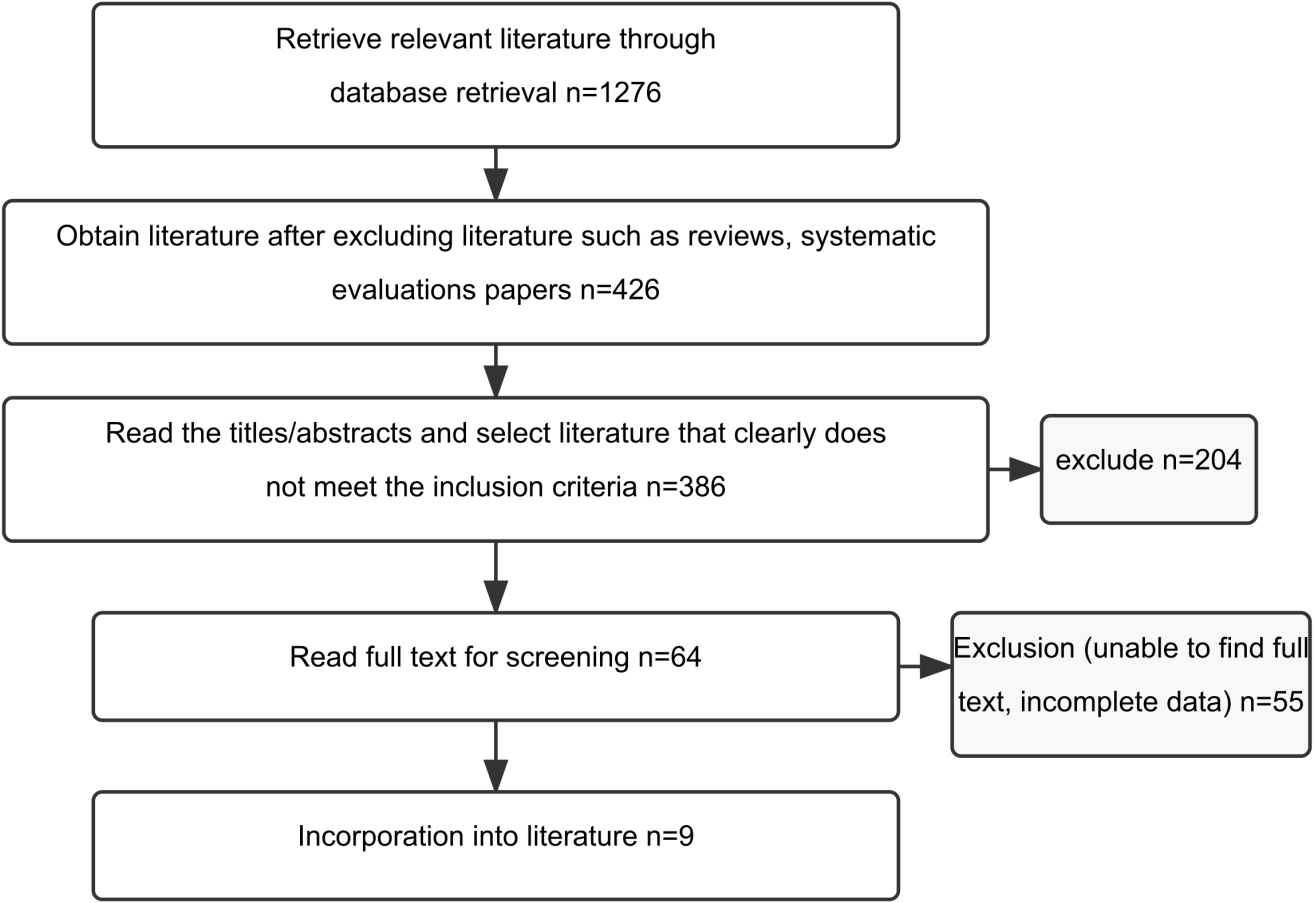
Literature screening process. This flowchart illustrates the systematic search strategy and selection process used to identify eligible studies for inclusion in the meta-analysis.

Our systematic search initially identified 1276 publications, which after applying rigorous inclusion/exclusion filtering, yielded 9 studies meeting all eligibility criteria (17–25). The collective participant pool encompassed 864 individuals, evenly distributed between experimental (n=432) and control (n=432) cohorts (**Figure 1**). Publication dates ranged from 2014 to 2023, with participant enrollment varying from 60 to 200 individuals per study. Demographic analysis revealed mean age distributions between 65 and 78 years with balanced gender representation. Disease severity classification showed predominance of mild-to-moderate AD (7/9 studies), while two investigations incorporated subjects across broader cognitive impairment spectrums. Intervention protocols consistently applied TCM rehabilitation nursing methodologies for experimental groups, while control populations received standard rehabilitation approaches. TCM interventions, though varying in specific composition, typically incorporated combinations of acupuncture therapy, massage techniques, emotional regulation strategies, TCM-based dietary guidance, and cognitive rehabilitation exercises. Intervention durations spanned 8 to 24 weeks, with 12-week protocols predominating. Treatment frequencies ranged from 3 to 7 weekly sessions, each lasting approximately 30–60 minutes. Comprehensive study characteristics appear in **Table 1**.

**Table 1:** Summary of the nine included studies.

Study (first-author, year)	Participants (n/groups, basic demographics)	Intervention (type & stated duration) vs. Control	Main outcome measures	Key result(s)
Zhang F 2016 (21)	60 AD patients (30 + 30); 56–85 yr, mean 70.5 ± 6.1 years; 17 M/13 F in TCM arm	*Traditional Chinese-medicine (TCM) nursing* added to routine care; 2-month cycles × 3 (≈ 6 months) vs. routine nursing	MMSE, ADL effective-rate	TCM nursing raised ADL responder rate to 80% (vs 53%) and MMSE responder rate to 77% (vs 20%).
Chen Y 2014 (24)	84 (42 + 42); 62–87 yr, mean 76.9 ± 5.8 yr; 27 M/15 F in TCM arm	Comprehensive TCM nursing package (emotional, diet, acupuncture, rehab) during 1–4 mo admission vs. basic nursing	“Overall clinical efficacy” composite	Total effectiveness 92.9% vs 78.6% in controls
Lu P 2015 (25)	60 (30 + 30); 61–83 yr, mean 64.6 ± 13.0 yr; 36 M/24 F	*Awakening-oriented* TCM “Xing-nao kai-qiao” nursing added to piracetam; duration not stated (study window 2010–2012) vs. piracetam alone	MMSE, CDT, MoCA	All three cognition scores improved significantly; higher overall efficacy P < 0.05
Zhang L 2018 (17)	62 (31 + 31); 57–84 yr (range)	*High-quality nursing* (holistic life-care, safety, communication) until discharge (length not reported) vs. routine nursing	MMSE, ADL	Post-care MMSE & ADL markedly higher in HQN arm P < 0.05
Ma Y 2018 (22)	200 (100 + 100); 58–75 yr, mean 65.8 ± 7.6 yr	High-quality nursing for 6 months (evaluated at 3 & 6 mo) vs. routine care	MMSE, MoCA, ADAS-cog, ADCS-ADL	HQN group showed higher MMSE/MoCA/ADL and lower ADAS-cog at both 3 & 6 mo, all P < 0.05
Zhu W 2022 (18)	98 (49 + 49); 61–77 yr, mean 66.5 ± 10.7 yr	*Multi-modal TCM nursing* (emotion, diet, acupuncture, rehab) during hospitalization vs. routine nursing	MMSE response; PedsQL QoL; treatment cooperation	MMSE Significant efficiency & all PedsQL domains improved; cooperation better, all P < 0.05
Luo Y 2021 (23)	100 (50 + 50); 62–88 yr, mean 73.5 ± 3.2 yr	*TCM “extended” nursing* (home-visit & follow-up) after discharge (duration not specified) vs. standard discharge advice	MMSE, ADL, generic QoL	Extended-nursing arm scored higher on MMSE, ADL, QoL (P < 0.05)
Qian P 2023 (19)	80 (40 + 40); mean 63 yr; balanced baseline	High-quality nursing programme (psychological support + daily-skills + cognitive training) during ward stay vs. routine nursing	MMSE, ADL, ADAS-cog, satisfaction	HQN improved MMSE & ADL and lowered ADAS-cog; satisfaction higher (all P < 0.05)
Shao S 2022 (20)	120 (60 + 60); 62–84 yr, mean ≈ 72 yr	*3R nursing model* (reminiscence, reality orientation, re-motivation) 30 min/day for 3 months vs. routine nursing	MMSE, ADL, GQOLI-74	After 3 mo, 3R arm outperformed controls on all three indices, P < 0.01

### 3.2 Quality assessment of included studies

Risk of bias evaluation was conducted independently by two reviewers using the Cochrane Collaboration’s assessment framework version 2.0, examining randomization procedures, allocation concealment mechanisms, blinding protocols for participants/personnel and outcome evaluators, outcome data completeness, selective reporting patterns, and additional bias sources. Evaluation disagreements were addressed through collaborative discussion or with third-reviewer adjudication. The assessment revealed that despite universal randomization claims, only four studies provided adequate randomization methodology descriptions. Allocation concealment remained undefined in most investigations (7/9). The hands-on nature of TCM interventions precluded participant/personnel blinding across all studies, though three investigations implemented outcome assessor blinding protocols. Data completeness and reporting transparency demonstrated low risk patterns throughout the study collection, suggesting reliable reporting practices for predetermined outcomes. Detailed RoB 2.0 assessments per study are summarized in **Table 2**.

**Table 2:** Cochrane Risk of Bias 2.0 (RoB 2.0) assessment for included studies.

Author	Year	Cochrane RoB 2.0 (overall)	Outcome indicator†	Cases (n)	Study group	Control group
Zhang L.P. et al. (17)	2018	High (randomisation & blinding not described)	②	31	31	
Zhu W.Y. (18)	2022	High (non-random two-arm nursing comparison)	①③④⑤	98	49	49
Qian P. (19)	2023	High (no details of sequence generation/masking)	②	80	40	40
Shao S.F. (20)	2022	High (3R-model vs routine care; no randomisation report)	②	120	60	60
Zhang F.J. (21)	2016	High (quasi-experimental, no concealment)	②③④⑤	60	30	30
Ma Y. et al. (22)	2018	High (no information on allocation/blinding)	②	200	100	100
Luo Y. (23)	2021	High (odd-even admission number grouping = quasi-random)	②	100	50	50
Chen Y.H. (24)	2014	Some concerns – random number table used but open-label; assessor blinding not reported	③④⑤	84	42	42
Lu P. (25)	2015	Some concerns – no concealment or blinding reported	①③④⑤	60	30	30

†Outcome indicators: ① MMSE ② ADL ③ Obvious effective rate ④ Effective rate ⑤ Ineffective rate.

### 3.3 Effects of TCM rehabilitation nursing on cognitive function

#### 3.3.1 MMSE score

**Figure 2:**
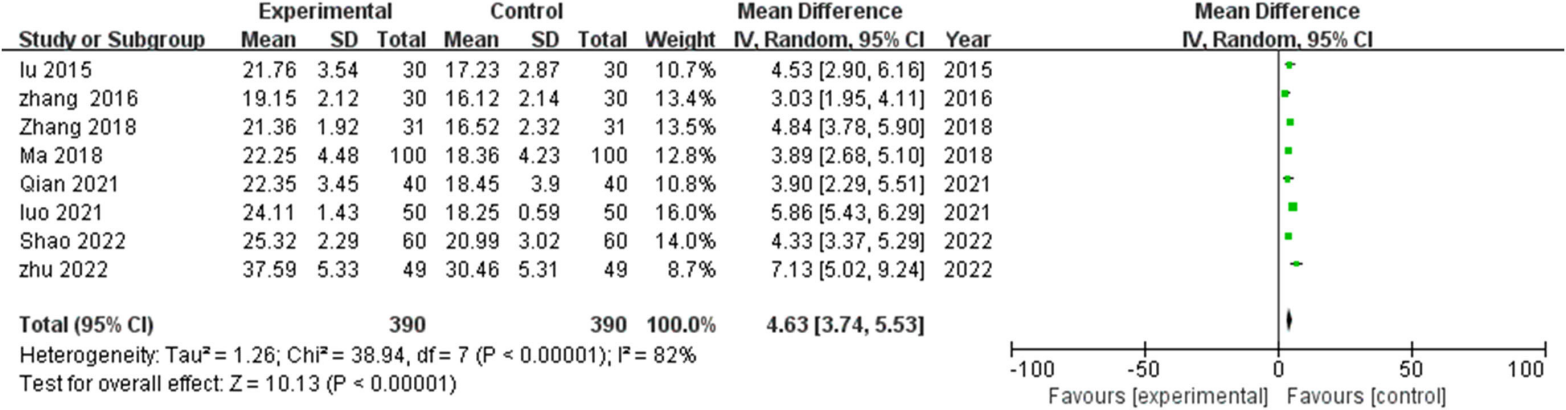
Meta-analysis of the impact of traditional Chinese medicine (TCM) rehabilitation nursing intervention on Mini-Mental State Examination (MMSE) scores in Alzheimer’s disease (AD) patients. The forest plot indicates a significant improvement in cognitive function with TCM rehabilitation nursing compared to conventional nursing care.

Examination of eight studies (17–23, 25) revealed significant cognitive enhancement following TCM rehabilitation nursing. Substantial inter-study heterogeneity (I^2^=82%, P<0.00001) necessitated random effects modeling. Meta-analytical synthesis demonstrated significantly elevated MMSE scores in TCM intervention recipients compared with controls (MD=4.63, 95% CI: 3.74-5.53, P<0.00001), indicating substantial cognitive function improvement (**Figure 2**).

Effect magnitude variations correlated with intervention duration, with most pronounced improvements observed in extended protocols (>12 weeks). The consistent positive direction of effect across all studies, with no negative outcome reports, strengthens confidence in TCM rehabilitation nursing’s beneficial impact on cognitive functioning in AD populations. To further explore heterogeneity (I²=82%), we conducted exploratory subgroup and sensitivity analyses using standardized mean differences (Hedges’ g) for the six studies (17, 19–22, 25) providing post-intervention means ± SDs. The pooled SMD was large and favorable (g=1.39, 95% CI 1.01–1.76, I²=74%). Subgroups by intervention duration showed large effects for both ≤12 weeks (k=1; g=1.61, 95% CI 1.19–2.02) and >12 weeks (k=3; g=1.16, 95% CI 0.78–1.55, I²=50%), with no significant between-group difference (QM not significant, low power). By TCM component, acupuncture-dominant (k=1; g=1.39, 95% CI 0.82–1.95) was comparable to multi-modal (k=5; g=1.40, 95% CI 0.96–1.84, I²=77%). Sensitivity analysis (leave-one-out) confirmed robustness, with pooled g ranging 1.24–1.51; no single study altered direction or significance, though Zhang 2018 had the largest influence (exclusion reduced g to 1.24).

#### 3.3.2 ADL score

**Figure 3:**
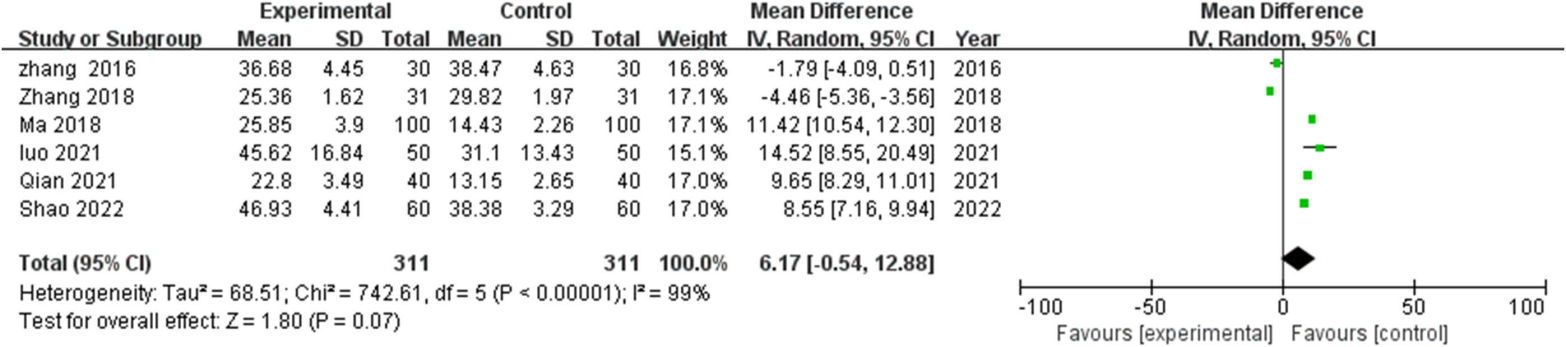
Meta-analysis of the impact of TCM rehabilitation nursing intervention on Activities of Daily Living (ADL) scores in AD patients. The analysis shows an improvement trend in ADL scores in the TCM group, though statistical significance was not achieved.

Analysis incorporating six studies (17, 19–23) examining functional independence demonstrated marked heterogeneity (I^2^=99%, P<0.00001), warranting random effects modeling. Though TCM intervention recipients demonstrated elevated ADL scores versus controls (MD=6.17, 95% CI: -0.54-12.88), this difference failed to achieve statistical significance (P=0.07) (**Figure 3**).

This finding suggests variable impact of TCM rehabilitation nursing on functional capabilities relative to its more consistent cognitive effects. The pronounced heterogeneity likely reflects methodological variations in functional assessment instruments, intervention intensity parameters, and baseline functional capabilities across study populations. A trend toward enhanced functional outcomes emerged in studies employing more intensive intervention protocols, though this pattern demonstrated inconsistency across the study collection. Exploratory SMD analysis on four studies (17, 19–21) yielded a very large effect (g=2.01, 95% CI 0.87–3.16, I²=94%). Subgroups: ≤12 weeks (k=1; g=2.18, 95% CI 1.73–2.64); >12 weeks (k=1; g=0.39, 95% CI -0.12–0.90). Acupuncture-dominant not assessable (no ADL data). Sensitivity: g varied 1.66–2.54; effects remained positive, with Zhang 2016 exerting downward influence.

### 3.4 Effects of TCM rehabilitation nursing on treatment efficacy

#### 3.4.1 Obvious effective rate

**Figure 4:**
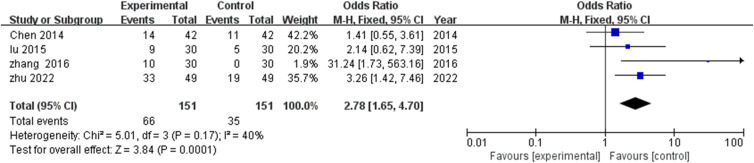
Meta-analysis of the impact of TCM rehabilitation nursing intervention on the significant efficiency rate in AD patients. The results indicate that TCM rehabilitation nursing significantly enhances the significant efficiency rate compared to the control group.

Four investigations (18, 21, 24, 25) documented marked clinical improvement rates. Analysis revealed modest heterogeneity (I^2^=40%, P=0.17), supporting fixed effects modeling application. TCM rehabilitation nursing significantly enhanced obvious effective rates compared with control interventions (MD=2.78, 95% CI: 1.65-4.70, P=0.0001), indicating nearly threefold greater likelihood of substantial clinical improvement among TCM recipients (**Figure 4**).

#### 3.4.2 Effective rate

**Figure 5:**
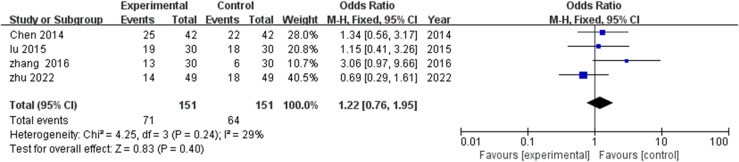
Meta-analysis of the impact of TCM rehabilitation nursing intervention on the effective rate in AD patients. The analysis suggests an improvement in the effective rate, though the difference between the groups was not statistically significant.

Evaluation of four studies (18, 21, 24, 25) examining moderate improvement rates demonstrated minimal heterogeneity (I^2^=29%, P=0.24), justifying fixed effects modeling. Despite numerically higher effective rates in TCM rehabilitation cohorts compared with controls (MD=1.22, 95% CI: 0.76-1.95), statistical significance was not achieved (P=0.40) (**Figure 5**).

This pattern suggests differential impact of TCM rehabilitation nursing across response categories, with pronounced effects on substantial improvement likelihood (obvious effective rate) but less distinctive advantage regarding moderate improvement outcomes. This profile may indicate particular benefit for optimizing recovery potential among intervention-responsive patients rather than producing uniform moderate improvements across heterogeneous AD populations.

#### 3.4.3 Inefficiency

**Figure 6:**
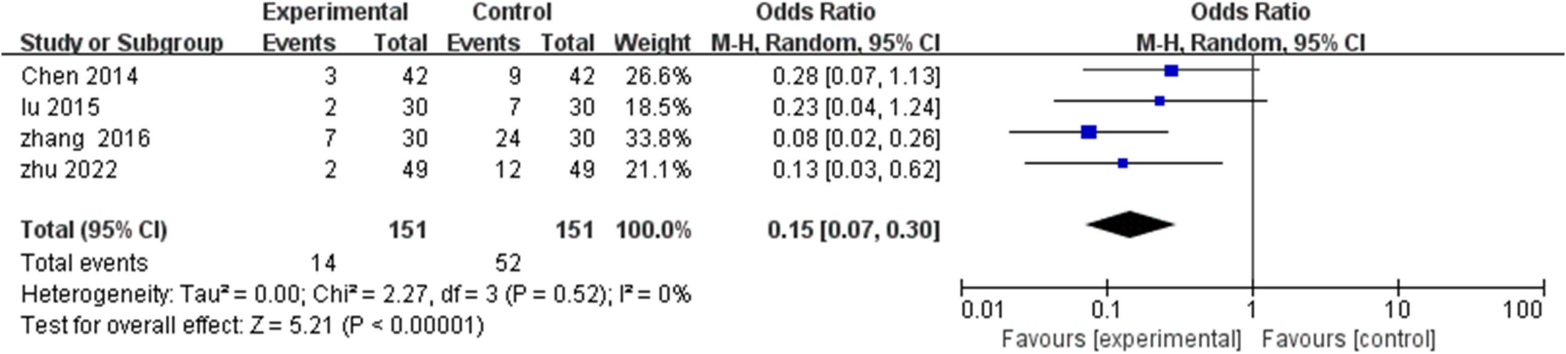
Meta-analysis of the impact of TCM rehabilitation nursing intervention on the inefficiency rate in AD patients. The findings demonstrate that TCM rehabilitation nursing reduces the inefficiency rate significantly compared to standard care.

Four studies (18, 21, 24, 25) documented treatment failure or minimal improvement rates. Negligible heterogeneity (I^2^=0%, P=0.52) supported fixed effects modeling application. TCM rehabilitation nursing significantly reduced inefficiency rates versus control interventions (MD=0.15, 95% CI: 0.07-0.30, P<0.00001) (**Figure 6**).

This substantial 85% reduction in treatment failure represents a clinically significant finding, indicating TCM rehabilitation nursing’s capacity to meaningfully reduce non-response proportions. Effect consistency across all four studies, despite protocol variations, enhances confidence in this therapeutic benefit.

### 3.5 Publication bias assessment

**Figure 7:**
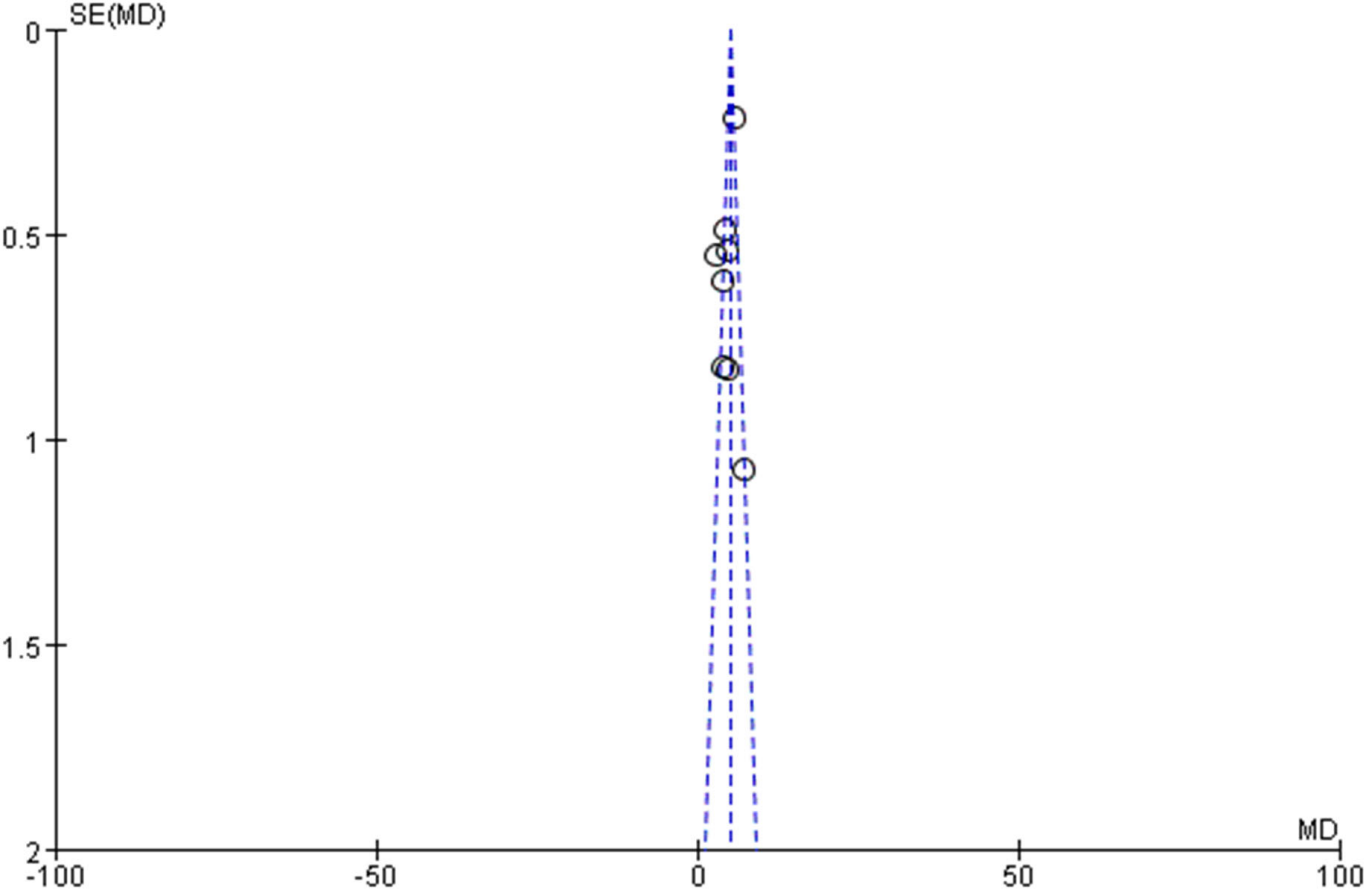
Funnel plot for publication bias assessment. This funnel plot evaluates potential publication bias in the included studies, revealing no significant asymmetry.

Publication bias evaluation through funnel plot visualization (**Figure 7**) revealed no significant asymmetry patterns, suggesting minimal publication bias within the included literature. Egger’s test results reinforced visual assessment findings (P=0.38), indicating low probability of publication bias influence on meta-analytical outcomes. However, the relatively limited study pool necessitates appropriate interpretive caution regarding potential undetected publication bias.

## 4 Discussion

This meta-analysis provides compelling evidence that TCM rehabilitation nursing interventions can significantly benefit cognitive function in patients with AD. The substantial improvement in MMSE scores (MD=4.63, 95% CI: 3.74-5.53) represents a clinically meaningful enhancement, particularly considering that a decline of 2–4 points annually is typical in untreated AD patients. This magnitude of improvement suggests that TCM rehabilitation approaches may target aspects of cognitive function through complementary pathways not addressed by conventional treatments. Furthermore, the marked reduction in treatment inefficiency rates (85% decrease compared to control interventions) indicates that TCM rehabilitation nursing substantially reduces the proportion of non-responders, a crucial consideration for clinical practice.

The differential effects observed between cognitive outcomes (MMSE) and functional measures (ADL) warrant careful interpretation. While cognitive improvements reached statistical significance, functional enhancements showed a positive trend without crossing the significance threshold. This pattern might reflect the complex relationship between cognitive ability and functional performance, where cognitive improvements may precede functional gains or require more intensive or prolonged intervention to manifest as measurable functional changes. Alternatively, the high heterogeneity observed in ADL measures (I²=99%) suggests substantial methodological variations in functional assessment that may have obscured treatment effects. The high heterogeneity (I²=82% for MMSE, 99% for ADL) likely arises from variations in intervention protocols (e.g., duration 3–6 months vs. unspecified; acupuncture vs. multi-modal), outcome scales (e.g., ADL directionality reversed in two studies), and baseline severity. Exploratory subgroups suggested shorter durations (≤12 weeks) may yield comparable or larger cognitive gains than longer ones, with no clear superiority of acupuncture-dominant approaches, though limited by small k. Sensitivity confirmed stable large effects (g>0.8), indicating clinical relevance despite variability—e.g., MMSE improvements exceed typical annual declines in AD.

AD presents with progressive memory deterioration and cognitive decline that significantly impact independence and quality of life. The main clinical manifestation of AD is memory loss, which progressively worsens as the disease advances. In severe cases, patients may lose their ability to function independently and perform basic self-care activities (26). Under normal circumstances, the nursing of AD patients presents significant challenges for their families. Most family caregivers lack professional nursing knowledge and tend to provide care based on experience or immediate patient needs. As the condition progresses, the family burden increases substantially, potentially compromising both patient recovery and family quality of life (27).

TCM rehabilitation nursing represents a multidimensional approach grounded in traditional Chinese medicine principles while incorporating contemporary rehabilitation science. This integrated methodology encompasses several key components: emotional regulation strategies that address psychological distress often accompanying cognitive decline; nutritional guidance based on TCM principles of food-medicine homology that supports neuroprotection; acupuncture and massage techniques theorized to improve cerebral circulation and remove harmful metabolites; and structured rehabilitation exercises designed to enhance both cognitive and physical functioning (28–30). Unlike compartmentalized Western approaches that often separate cognitive, physical, and emotional interventions, TCM rehabilitation nursing applies a systems-based perspective that views these domains as inherently interconnected, potentially addressing multiple pathophysiological mechanisms simultaneously.

Compared with other non-pharmacological approaches such as cognitive stimulation therapy, reminiscence therapy, and physical exercise programs, TCM rehabilitation nursing appears to offer a more comprehensive treatment paradigm. While these other approaches typically target isolated domains of functioning, TCM rehabilitation nursing integrates multiple therapeutic modalities into a coherent system. The superior efficacy in reducing inefficiency rates suggests this integrated approach may reach patients who might not respond to more narrowly focused interventions.

The implementation model combining inpatient TCM nursing with continued home-based care represents a pragmatic approach to long-term management. This continuity of care through family involvement promotes sustainable outcomes beyond the clinical setting. A combination of inpatient TCM nursing and home nursing is typically employed, with emotional nursing, acupuncture, massage, and rehabilitation training emphasized during hospitalization. After discharge, family members continue care according to medical guidance, implementing dietary control, daily life management, rehabilitation training, and massage techniques that maximize the advantages of TCM rehabilitation nursing, significantly improving patients’ cognitive abilities, correcting maladaptive behaviors, enhancing nursing compliance, and improving overall quality of life (23).

Rehabilitation training constitutes a critical component of the TCM nursing intervention for AD patients. Effective rehabilitation protocols appear to share certain characteristics across the studies analyzed: progressive difficulty calibration to maintain appropriate cognitive challenge; personalization based on individual cognitive profiles and functional needs; integration of meaningful life activities rather than abstract exercises; and consistent positive reinforcement to maintain motivation and engagement. Rehabilitation training after discharge represents a particularly important measure in nursing intervention for AD patients. Effective training significantly mobilizes patient initiative, encouraging completion of established movements while providing induced health education, language training, and self-care instruction, all of which foster enthusiasm for recovery and create greater possibilities for improvement (31).

From a health economics perspective, TCM rehabilitation nursing may offer advantages in resource-limited settings. The equipment required for TCM rehabilitation nursing is simple, economical, easy to learn, easy to operate, and readily disseminated, giving it high promotion value (32). Additionally, psychological nursing of patients is essential during cognitive training processes. Nursing staff should emphasize humanistic care, alleviating tension, anxiety, depression, and other negative emotions to improve treatment compliance (33).

While all included studies were conducted in China, TCM has been increasingly adopted as a complementary medical approach in many parts of the world. However, cultural acceptance of TCM may influence treatment response. It would be enriching to reflect on how the positive cultural predisposition toward TCM in China could amplify the observed effects—possibly through placebo mechanisms or increased adherence to the intervention.

Several limitations of this meta-analysis warrant consideration. First, the relatively small number of included studies limits the robustness of subgroup analyses that might identify optimal intervention parameters or patient characteristics associated with maximal benefit. Second, methodological heterogeneity across studies, particularly regarding intervention protocols and outcome measurements, introduces uncertainty in effect size estimation. Third, the included studies lacked standardized long-term follow-up, leaving questions about the durability of observed benefits unresolved. Additionally, most studies were conducted in Chinese populations, potentially limiting generalizability to other ethnic and cultural contexts where different healthcare beliefs and practices may influence intervention acceptability and effectiveness. Furthermore, the challenges of implementing appropriate blinding procedures in rehabilitation interventions introduce potential performance and detection biases that cannot be completely eliminated. Additionally, quantitative exploration was limited to studies with full means/SDs (6/9 for MMSE, 4/9 for ADL), excluding three due to incomplete reporting. Small subgroups reduced power for moderator tests; future trials should standardize outcomes and protocols.

Future research should address these limitations through several approaches. First, larger multi-center randomized controlled trials with standardized intervention protocols would strengthen the evidence base and facilitate more precise effect size estimation. Second, studies should incorporate longer follow-up periods (minimum 12 months) to assess durability of effects and potential impact on disease progression. Third, investigation of dose-response relationships and identification of critical intervention components would support optimization of clinical protocols. Fourth, exploration of potential mechanisms through biomarker assessment would enhance understanding of therapeutic pathways. Finally, studies examining TCM rehabilitation nursing in diverse populations and in combination with standard pharmacological treatments would inform clinical integration strategies.

In conclusion, this meta-analysis demonstrates that TCM rehabilitation nursing interventions significantly improve cognitive function in AD patients and substantially reduce treatment failure rates. While the effects on activities of daily living require further investigation, the overall evidence supports the integration of TCM rehabilitation nursing approaches into comprehensive care plans for individuals with AD. The combined inpatient and home-based implementation model offers a practical framework for sustainable intervention delivery that merits wider clinical adoption and continued research refinement.

## Data Availability

The raw data supporting the conclusions of this article will be made available by the authors, without undue reservation.
