# Supplementary material for: Cognitive function enhancement in Alzheimer’s disease through traditional Chinese medicine rehabilitation nursing: meta-analysis
Source: Front Psychiatry. 2025 Aug 13;16:1631589. doi: 10.3389/fpsyt.2025.1631589 (PMC12380736; doi:10.3389/fpsyt.2025.1631589)
Supplement: Supplementary file 1 [file DataSheet1.docx]

**Full Search Strategies:**

PubMed: ("Alzheimer Disease"[Mesh] OR Alzheimer* OR dementia) AND ("Medicine, Chinese Traditional"[Mesh] OR TCM OR "Traditional Chinese Medicine") AND (rehabilitation OR nursing OR intervention) Filters: from 2010 - present Embase: ('alzheimer disease'/exp OR alzheimer*:ab,ti OR dementia:ab,ti) AND ('traditional chinese medicine'/exp OR tcm:ab,ti OR 'chinese medicine':ab,ti) AND (rehabilitation:ab,ti OR nursing:ab,ti OR intervention:ab,ti) AND [2010-2025]/py CNKI: (SU='阿尔茨海默病' OR SU='痴呆') AND (SU='中医' OR SU='中药' OR SU='传统中医') AND (SU='康复护理' OR SU='护理干预') Year:2010-2025 Similar for Wanfang and VIP.
